# Supplementary material for: Chromothripsis during telomere crisis is independent of NHEJ, and consistent with a replicative origin
Source: Genome Res. 2019 May;29(5):737–49. doi: 10.1101/gr.240705.118 (PMC6499312; doi:10.1101/gr.240705.118)
Supplement: Supplemental Material [file supp_gr.240705.118_Supplemental_file_1.zip › contigs/annotated_contigs/DB57/contig.2.DB57_length_558_mean_cov_4.8082437276.docx]

**DB57_length_558_mean_cov_4.8082437276**

```
TCTCACTCTGGAGAGCTGTGGCACAAAAATAGCTCACTGCAGCCTTGGACTCCTGGGTTCAAGTGAATCTCCCATGTCAGCCTCCCGAG
 >chr7:140280158-140280499 + E=1e-190
TAGTTAGGACTACAGGCATGCACCACCATGCCTATCTAATTTTTAAATTTTTTATAGAGATGGGGTCTTACTATGTTGACCAGGCTGGT

CTTGAATGCCTGGCCTCAAGCAATCCTCCCACCTTGGCTTCCCAAAGTGCTAGGATTACAGGCATGGACCACTGCACCCAGCCTGTTAT

TTTACTATTATTATTATTTATTTATTTTTGAGATGGAGTCTCATTCTGTTGCCCAGGCTGGAGTGTAGTGGTGC|GCCCGGCCTCTTAT
                                                                            >chr7:140288
ACACATTTTATTTATCAGAAGACAGAGGCATAGAGGGGTTAGATAAATTGCTCCAGGTATATTGGGAAAAGGTGGTGGAATTAGGGTCT
834-140289051 + E=6e-119
GACCGCAGGAAGTCTGGTTGGAGACTGATGCTTTTAATGACACAGGGCATTATGGAGAGCATTTATTTAAGTGAGGCATGTCAGGAGGG

AAGCTGCATTCACAGGCTCCAAACT
```
